# Pulmonary embolism presenting as syncope: a case report

**DOI:** 10.4076/1752-1947-3-7440

**Published:** 2009-09-15

**Authors:** Ahmet Demircan, Gulbin Aygencel, Ayfer Keles, Ozgur Ozsoylar, Fikret Bildik

**Affiliations:** 1 Department of Emergency Medicine, Gazi University Faculty of Medicine, Ankara, Turkey; 2 Department of Internal Medicine, Gazi University Faculty of Medicine, Ankara, Turkey; 3 Department of Anesthesiology and Reanimation, Gazi University Faculty of Medicine, Ankara, Turkey

## Abstract

**Introduction:**

Despite the high incidence of pulmonary embolism its diagnosis continues to be difficult, primarily because of the vagaries of symptoms and signs in presentation. Conversely, syncope is a relatively easy clinical symptom to detect, but has varied etiologies that lead to a documented cause in only 58% of syncopal events. Syncope as the presenting symptom of pulmonary embolism has proven to be a difficult clinical correlation to make.

**Case presentation:**

We present the case of a 26-year-old Caucasian man with pulmonary embolism induced-syncope and review the pathophysiology and diagnostic considerations.

**Conclusions:**

Pulmonary embolism should be considered in the differential diagnosis of every syncopal event that presents at an emergency department.

## Introduction

Recognized venous thromboembolism (pulmonary embolism and deep venous thrombosis) is responsible for more than 250,000 hospitalizations and approximately 50,000 deaths per year in the United States. Because it is difficult to diagnose, the true incidence of pulmonary embolism is unknown, but it is estimated that approximately 650,000 cases occur annually [1].

Despite this high incidence, the diagnosis of pulmonary embolism continues to be difficult primarily because of the notorious vagaries of symptoms and signs in its presentation. Conversely, syncope is a relatively easy clinical symptom to detect, but has varied etiologies that lead to a documented cause in only 58% of syncopal events [2].

Syncope as the presenting symptom of pulmonary embolism has proven to be a difficult clinical correlation to make. We present the case of a patient with pulmonary embolism-induced syncope and review the pathophysiology and diagnostic considerations.

## Case presentation

A 26-year-old Caucasian man with no history of disease was admitted to Gazi University Emergency Department after he had a syncopal episode in his home. The patient was in his usual good state of health until he suddenly collapsed while standing and lost consciousness for approximately five minutes. He recovered spontaneously but was extremely weak and dyspneic. He was also diaphoretic and tachypneic, but denied any associated chest pain or palpitations. No tonic-clonic activity was witnessed, and he experienced no incontinence.

The patient was a computer programmer and he had been working 18 hours a day without rest periods for a month. On admission, physical examination revealed a diaphoretic and dyspneic patient without focal neurologic findings. His heart rate was regular but tachycardic at 128 beats/minute, his blood pressure was 126/72 mmHg without orthostatic changes, and his respiratory rate was 32 breaths/minute. The room air oxygen saturation was 90%, and arterial blood gas analysis in room air revealed hypoxemia (PO_2_ = 58 mmHg) with an elevated alveolo-arterial oxygen gradient (A-a O_2_ gradient). Examination of his head and neck was normal. The results of chest wall examination revealed reduced breath sounds bilaterally at the lung bases. The findings of heart and abdominal examinations were unremarkable, but on examination of his legs, deep venous thrombosis (DVT) was noted in his left leg, with a positive Homans' sign in the left leg and the left calf measured 3 cm more than the right one.

**Figure 1:**
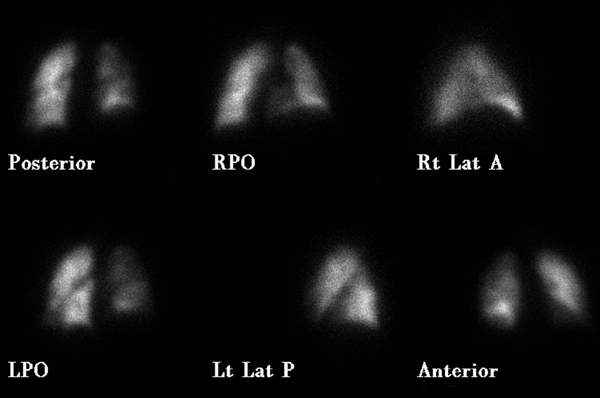
**Decreased perfusion is seen to the right lung (particularly evident in the right lower lobe on the RPO image) in our case (perfusion scan was performed with Tc-99m MAA)**.

**Figure 2:**
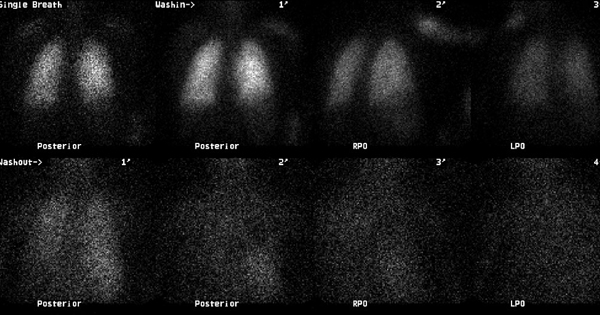
**There is no significant ventilation defect in our case (ventilation scan was performed with Xe-133 gas)**.

Levels of serum electrolytes, glucose, blood urea and creatinine, and complete blood counts were normal. Results of a computed tomographic scan of his head were negative for bleeding, aneurysm or an embolic event. Chest X-ray was clear. An electrocardiogram showed a regular rhythm consistent with sinus tachycardia; there were Q and T waves in lead III and an S wave in lead I. A ventilation-perfusion scan demonstrated an unmatched segmental perfusion defect, indicating a high probability of the presence of a pulmonary thromboembolism (PTE) (Figures 1 and 2). A transthoracic echocardiogram revealed normal left ventricle function without a patent foramen ovale, an atrial septal defect or a ventricular septal defect, but with mild pulmonary hypertension (42 mmHg). A Doppler scan of the legs revealed an acute DVT in the patient's left leg, in the popliteal vein. Thrombolytic treatment was not given - the patient received standard anticoagulation treatment with unfractionated heparin and an oral anticoagulant. Before treatment, a blood sample was taken to examine the thrombophilia panel. After a 12-day course of hospital treatment, he was discharged on oral warfarin therapy. The patient's long-term follow-up was performed by the Department of Pulmonary Disease, and we learned that the patient was well for four months after that episode without any evidence of recurrent syncope or pulmonary embolism.

## Discussion

Pulmonary embolism is a frequent cause of death in the United States. Nevertheless, it remains difficult to diagnose. Pulmonary emboli differ considerably in size and number, and the underlying disorders, including malignancy, trauma, and protein C or S deficiency, are numerous [1]. The classic triad of pleuritic chest pain, dyspnea, and hemoptysis is rare, and clinically apparent DVT is present in only 11% of confirmed cases of pulmonary embolism in patients without underlying cardiopulmonary disease [3].

However, the clinical picture of pulmonary embolism is variable and most patients suffering from acute pulmonary embolism present with one of three different clinical syndromes. These clinical syndromes are pulmonary infarction, acute unexplained dyspnea, and acute cor pulmonale. The pulmonary infarct syndrome usually occurs with a submassive embolism that completely occludes a distal branch of the pulmonary circulation. Patients with this condition have pleuritic chest pain, hemoptysis, rales, and abnormal findings on chest X-ray. The acute, unexplained dyspnea pattern may also be the result of submassive pulmonary embolism without pulmonary infarction. Results of a chest X-ray and electrocardiogram are usually normal, but pulse oxygen saturation is often depressed. The third pattern, acute cor pulmonale syndrome, is caused by the complete obstruction of 60 to 75% of pulmonary circulation. Patients with this pattern experience shock, syncope, or sudden death [4,5].

Syncope, in contrast to pulmonary embolism, is relatively easy to detect, but can be a difficult symptom from which to determine the etiology. In as many as 50% of patients with syncope, no specific cause is found despite extensive evaluation. Syncope has been classified as cardiovascular (reflex and cardiac syncope), noncardiovascular (including neurologic and metabolic disorders) and unexplained [2,6]. It occurs in approximately 10% of patients with acute pulmonary embolism and is commonly ascribed to a massive, hemodynamically unstable acute pulmonary embolism. Although the prognostic value of syncope has not been specifically addressed, it has generally been considered a poor indicator in diagnosing pulmonary embolism [7].

Syncope in the setting of pulmonary embolism can be the result of three possible mechanisms. First, greater than 50% occlusion of the pulmonary vascular tree causes right ventricular failure and impaired left ventricular filling, leading to a reduction in cardiac output, arterial hypotension, reduced cerebral blood flow, and ultimately syncope. The second mechanism of syncope associated with pulmonary embolism is the appearance of arrhythmias associated with right ventricular overload. In the third mechanism, the embolism can trigger a vasovagal reflex that leads to neurogenic syncope. However, the contribution of hypoxemia secondary to ventilation or perfusion abnormalities must also be considered and may play an important role in the development of syncope. Moreover, acute pulmonary hypertension may also lead to right-to-left flow across a patent foramen ovale, and thus exacerbate hypoxemia [8,9].

The clinician should seek the following clues to the diagnosis of pulmonary embolism in patients who have had a syncopal episode: (a) hypotension and tachycardia or transient bradyarrhythmia; (b) acute cor pulmonale according to electrocardiogram criteria or physical examination; and (c) other signs and symptoms indicative of pulmonary embolism. The presence of any of these findings without other obvious causes of syncope should lead to further work-up, including arterial blood gas analysis, ventilation-perfusion scanning, lower extremity duplex sonogram, echocardiography, multislice computed tomography and angiography, if necessary. Although oxygen saturation levels are inadequate for screening purposes, respiratory alkalosis with hypoxia and increased A-a O_2_ gradient are typically seen. However, results of blood gas analysis are normal in 10% of cases [4,10].

In our case, the patient presented to the emergency department with complaints of dyspnea, tachypnea and tachycardia, following a syncopal episode. He had experienced immobilization for one month, hypoxemia in room air, and DVT according to the ultrasonographic results. PTE was initially considered and all of the diagnostic procedures were carried out to prove this presumptive diagnosis. Because DVT and PTE developed in this young patient with no history of any underlying diseases or disorders, he was referred for thrombophilia panel testing (including protein C or S deficiency and Factor V mutation) before treatment; however, as his long-term follow-up was performed by the Department of Pulmonary Diseases, we do not have any further detailed results from these examinations. This case is interesting because the patient did not experience a massive embolism but did develop syncope.

## Conclusion

Pulmonary embolism presenting with syncope is difficult to diagnose. Physicians and other health care professionals must be vigilant with patients who have syncope, because this symptom may be a 'forgotten sign' of life-threatening pulmonary embolism.

## Consent

Written informed consent was obtained from the patient for publication of this case report and any accompanying images. A copy of the written consent is available for review by the Editor-in-Chief of this journal.

## Competing interests

The authors declare that they have no competing interests.

## Authors' contributions

AD, AK and FB analyzed and interpreted the patient data regarding the syncope and the pulmonary embolism. GA and OO performed the acute treatment of the patient, and were major contributors in writing the manuscript. All authors read and approved the final manuscript.
